# Operative vs. Nonoperative Management of Isolated Weber B Ankle Fractures

**DOI:** 10.7759/cureus.78028

**Published:** 2025-01-26

**Authors:** Hamza K Toru, Asad Ali Khan, Nasir Ali

**Affiliations:** 1 Orthopedics, Khyber Teaching Hospital, Peshawar, PAK; 2 Orthopedics, Saidu Teaching Hospital, Swat, PAK; 3 Orthopedics, Qazi Hussain Ahmad Medical Complex, Nowshehra, PAK

**Keywords:** ankle fractures, complications, functional recovery, nonoperative management, operative management, patient satisfaction, weber b fractures

## Abstract

Background and objective

Ankle fractures (AFs) are common in orthopedic practice, with Weber B fractures representing a significant proportion. These fractures occur at the syndesmotic level of the fibula and can be treated either operatively or nonoperatively. However, the optimal management approach remains debated, particularly for isolated fractures without medial or syndesmotic involvement.

This study aims to compare the operative and nonoperative management outcomes of isolated Weber B AFs in terms of functional recovery, complication rates, and patient satisfaction.

Methodology

This retrospective cohort study was conducted at the Department of Orthopedics, Hayatabad Medical Complex, Peshawar, from February 3, 2022, to March 2, 2023. A total of 115 patients with isolated Weber B AFs were included. Patients were categorized into operative (*n* = 65, 56.5%) and nonoperative (*n* = 50, 43.5%) groups. Outcomes assessed at six months included functional recovery using the Olerud-Molander Ankle Score (OMAS), complication rates (e.g., malunion, nonunion, and infection), and patient satisfaction levels. Statistical analyses were performed to compare outcomes between groups.

Results

The operative group demonstrated significantly higher OMASs (89.2 ± 6.3) than the nonoperative group (81.4 ± 8.1, *P* = 0.009). Malunion occurred in 4 patients (8%) in the nonoperative group but was not observed in the operative group (*P* = 0.034). Surgical site infections were observed in 3 patients (4.6%) in the operative group. Patient satisfaction was higher in the operative group, with 49 patients (75.3%) reporting being *very satisfied *compared to 32 patients (64%) in the nonoperative group (*P* = 0.045).

Conclusions

Operative management of isolated Weber B fractures offers better functional recovery and a lower risk of malunion compared to nonoperative treatment. For stable fractures, nonoperative management remains a valid option. Further long-term studies are required to assess the durability of outcomes for both approaches.

## Introduction

Ankle fractures (AFs) are one of the most common types of injuries managed in orthopedic practice, representing approximately 9% of all fractures in adults. Of these, Weber B fractures, which involve the fibula at the level of the syndesmosis, form a distinct subset. They are commonly sustained through low-energy trauma caused by twisting mechanisms often encountered in sports and daily life [1,2]. The treatment of Weber B fractures, especially those not involving an associated injury of the medial structures or syndesmosis is controversial [3].

Delayed ankle mobilization through the use of either a cast or a boot and surgical treatment via open reduction and internal fixation (ORIF) has had various advocates in the management of isolated Weber B fractures. Supporting surgical treatment rationales that surgical treatment provides better anatomical reduction and stability, leading to decreased malunion and related post-traumatic arthritis [4,5]. Conversely, proponents of nonoperative management highlight its benefits, including the avoidance of surgical risks, lower costs, and comparable functional outcomes in selected cases [6,7].

Several factors affect the decision-making process when it comes to isolated Weber B fractures for treatment, such as displacement, stability of the fracture, and patient-specific factors, including activity level, comorbidities, and preferences [8]. Although surgery might be necessary in dislocated or unstable fractures, the utilization of nonoperative treatment is becoming more known in stable fractures. Recent developments in imaging modalities such as weight-bearing radiographs and stress testing have allowed for a more granular characterization of fracture stability, which, in turn, directs surgical treatment algorithms [9].

The quest for defining the criteria of surgical intervention in Weber B fractures has been a hot topic in recent years, and published literature has challenged the dogma that all Weber B fractures require surgical intervention [10] and highlighted the importance of fracture stability and patient-specific parameters. Additionally, the advantages of nonoperative techniques have been established in target populations, providing evidence that is comparable to, or even superior to, surgery, thereby challenging the well-established paradigm of surgery as the gold standard [11].

This study seeks to assess and compare the operative vs. nonoperative treatment outcomes of isolated Weber B fractures. We aim to participate in the ongoing dialogue regarding how to best treat these injuries by reporting functional outcomes, complications, and patient satisfaction.

## Materials and methods

This retrospective cohort study was conducted at the Department of Orthopedics, Hayatabad Medical Complex, Peshawar, for a duration of one year (February 3, 2022, to March 2, 2023). Ethics approval for data collection was granted by the Institutional Review Board (IRB) before the start of the study, under reference number no. 458/ADR/KMC

The cohort consisted of 115 patients diagnosed with isolated Weber B AFs - fibular fractures at the syndesmotic level, without associated medial malleolus or syndesmotic ligament injuries confirmed by radiological examination.

The inclusion criteria for the study were adults aged 18 years or older with isolated Weber B fractures, classified as either stable or unstable. The stability of the fracture was assessed through a combination of the following:

(1) Radiographic examination: Fracture displacement and congruence of the ankle joint were reviewed using weight-bearing radiographs where applicable.

(2) Stress testing: Stability was further confirmed through stress radiographs (e.g., external rotation stress views) or intraoperative assessment in cases requiring operative management. Patients with stable fractures were included in the nonoperative group, while those with unstable fractures were treated operatively. Stability was thus a key determinant variable for deciding the treatment modality.

The exclusion criteria included associated medial malleolus fractures or syndesmotic injuries requiring stabilization, open fractures or fractures associated with polytrauma, and patients with incomplete medical records or follow-up data. Data were extracted from hospital records, including demographics (age and gender), clinical presentation, radiological findings, treatment modality (operative vs. nonoperative), and posttreatment outcomes. Fracture pattern, displacement, and stability were confirmed by reviewing radiographs.

Operative management (*n* = 65)

This group of patients was treated with ORIF using a routine surgical technique. Intraoperative stress testing was used to determine stability, with syndesmotic screw fixation performed as needed.

Nonoperative management (*n *= 50)

Nonoperative management was implemented for patients with fractures deemed stable based on radiographic findings and intraoperative or clinical stress tests. Immobilization was achieved using either a below-knee plaster cast or a functional brace. The immobilization period ranged from four to six weeks, and patients were encouraged to bear weight early, depending on radiographic evidence of fracture healing and stability. Regular follow-ups were conducted, including clinical and radiographic evaluations, to monitor progress.

Outcome assessment

Primary Outcomes

Functional outcomes were assessed using the OMAS at six months post-intervention. OMAS is a validated self-administered questionnaire evaluating pain, stiffness, swelling, stair climbing, running, and work/activities. Each parameter is scored, and the total score ranges from 0 to 100, with higher scores indicating better function: 91-100, Excellent; 61-90, Good; 31-60, Fair; 0-30, Poor.

Secondary Outcomes

Secondary outcomes included complications (malunion, nonunion, post-traumatic arthritis, and infection) and patient-reported satisfaction. Satisfaction was assessed using a three-point Likert scale (satisfied, neutral, dissatisfied).

Data analysis was performed using SPSS version 25.0 (IBM Corp., Armonk, NY). Continuous variables, such as OMASs, were summarized as mean ± standard deviation, while categorical variables, including complications and patient satisfaction, were presented as frequencies and percentages. Fracture stability (classified as stable vs. unstable) was included as a covariate in the analysis to evaluate its influence on treatment outcomes. Comparative analyses were conducted between the operative and nonoperative groups, with subgroup analyses performed to account for fracture stability. The independent sample t-test was used to compare continuous variables (OMAS) between the two groups, and the chi-square test was employed to assess differences in categorical variables (complications and patient satisfaction). Logistic regression analysis was performed to adjust for potential confounders, including age, fracture stability, and other baseline characteristics. A *P*-value < 0.05 was considered statistically significant.

## Results

A total of 115 patients with isolated Weber B AFs were included in this study. The mean age was 42.8 ± 12.7 years (range: 18-68 years), with a nearly equal gender distribution (males: 55 patients, 47.8%; females: 60 patients, 52.2%). The operative group included 65 patients (56.5%), while the nonoperative group comprised 50 patients (43.5%). The average body mass index (BMI) was 27.3 ± 4.5 in the operative group and 26.8 ± 4.2 in the nonoperative group, with no significant difference between the groups (*P* = 0.421). Comorbidities were present in 40 (34.8%) patients, with 22 patients (33.8%) in the operative group and 18 patients (36%) in the nonoperative group, showing no significant difference (*P* = 0.756). The smoking rate was similar between the groups, with 18 (27.7%) smokers in the operative group and 15 (30%) in the nonoperative group, with no statistically significant difference (*P* = 0.686) (Table 1).

**Table 1:** Baseline demographics of the study population. Statistical tests used: Chi-square test for categorical data and independent t-test for continuous data.

Characteristics	Operative group	Nonoperative group	Total	*P*-value
Mean age (years)	43.1 ± 12.9	42.4 ± 12.4	42.8 ± 12.7	0.732
Age range (years)	18-68	19-67	18-68	
Males	28 (43.1%)	27 (54%)	55 (47.8%)	0.256
Females	37 (56.9%)	23 (46%)	60 (52.2%)	
Body mass index (BMI)	27.3 ± 4.5	26.8 ± 4.2	27.0 ± 4.4	0.421
Comorbidity (%)	22 (33.8%)	18 (36%)	40 (34.8%)	0.756
Smoking status (%)	18 (27.7%)	15 (30%)	33 (28.7%)	0.686

Functional recovery was assessed using the OMASs at six months posttreatment. Patients in the operative group achieved statistically significantly higher OMASs (89.2 ± 6.3) than those in the nonoperative group (81.4 ± 8.1) (Table 2).

**Table 2:** Functional outcomes based on the Olerud-Molander Ankle Score (OMAS) at six-month follow-up. OMAS ≥ 80 indicates good to excellent functional recovery. Statistical tests used: Independent t-test and chi-square test.

Functional recovery	Mean OMAS	Patients achieving ≥ 80 OMAS	*P*-value
Operative group (*n* = 65)	89.2 ± 6.3	60 (92%)	0.009
Nonoperative group (*n* = 50)	81.4 ± 8.1	37 (74%)	

The comparison of complications between the groups highlights notable differences. Malunion occurred in 4 patients (8%) in the nonoperative group, while no malunion was seen in the operative group (*P* = 0.034). Nonunion was not observed in either group, indicating effective management of this complication with both approaches. Post-traumatic arthritis was rare, affecting 1 patient (1.5%) in the operative group and 1 (2%) patient in the nonoperative group, with no significant difference (*P* = 0.786). Surgical site infections were noted exclusively in the operative group, affecting 3 patients (4.6%), while syndesmotic malreduction was observed in 1 patient (1.5%) in the same group; both did not apply to the nonoperative group (Table 3).

**Table 3:** Comparison of complications between operative and nonoperative groups. Statistical test used: Chi-square test. NA: Not applicable (as these complications occur only in the operative group). -: *P*-value not calculated due to the absence of events in both groups.

Complications	Operative group	Nonoperative group	*P*-value
Malunion	0 (0%)	4 (8%)	0.034
Post-traumatic arthritis	1 (1.5%)	1 (2%)	0.786
Nonunion	0 (0%)	0 (0%)	-
Surgical site infection	3 (4.6%)	NA	-
Syndesmotic malreduction	1 (1.5%)	NA	-

Among the participants, 49 patients (75.3%) in the operative group reported being *very satisfied* with their treatment, compared to 32 (64%) in the nonoperative group. In terms of *satisfied* patients, 12 patients (18.4%) of the operative group and 11 patients (22%) of the nonoperative group reported satisfaction. Three patients (4.6%) of the operative group and 5 patients (10%) of the nonoperative group were neutral about their outcomes. Additionally, 1 patient (1.5%) of the operative group and 2 patients (4%) of the nonoperative group were *dissatisfied *(Table 4).

**Table 4:** Patient satisfaction with treatment at six-month follow-up. Statistical test used: Chi-square test.

Satisfaction level	Operative group, *n* (%)	Nonoperative group, *n* (%)	*P*-value
Satisfied	61 (93.7%)	43 (86%)	0.045
Neutral	3 (4.6%)	5 (10%)	0.091
Dissatisfied	1 (1.5%)	2 (4%)	0.432

**Figure 1:**
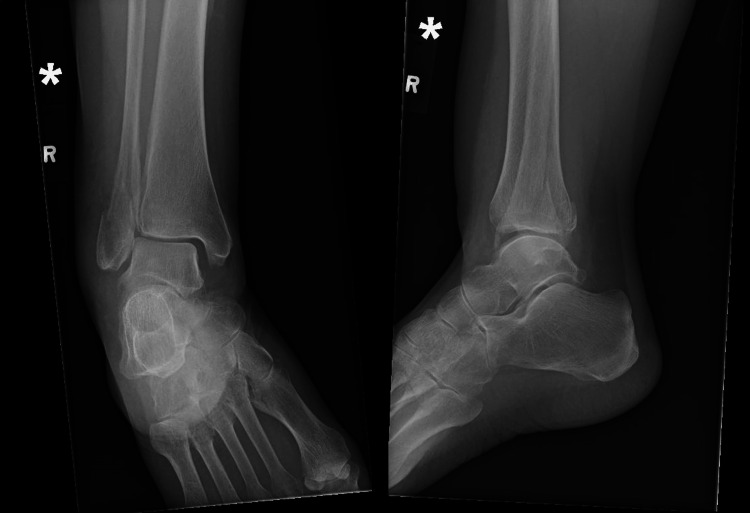
Preoperative Weber B fracture.

**Figure 2:**
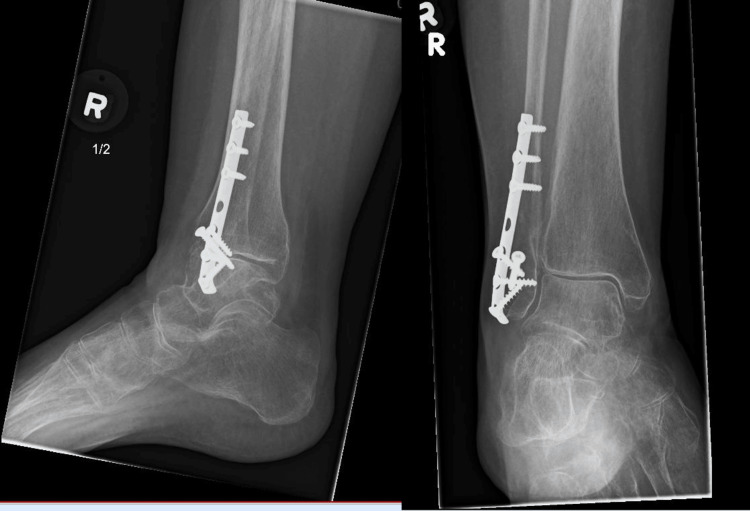
Postoperative X-ray of Weber B fracture.

Radiographic stability at three and six months posttreatment showed that 64 patients (98.5%) in the operative group achieved and maintained proper anatomical alignment, demonstrating the effectiveness of the surgical intervention. In contrast, 4 patients (8%) in the nonoperative group exhibited slight displacement, leading to minor malunion, although none of these patients experienced any functional impairment (Figures 1, 2; Table 5).

**Table 5:** Radiographic outcomes at three and six months posttreatment.

Group	Radiographic outcome	Number (*n*)	Percentage (%)
Operative group	Achieved better anatomical alignment and maintained reduction	64	98.5%
Nonoperative group	Slight displacement with minor malunion, no functional impairment	4	8%

## Discussion

AFs, especially Weber B fractures, are common injuries, and their treatment remains subject to ongoing debate in orthopedic practice. This study aimed to compare the operative versus nonoperative treatment outcomes of isolated Weber B fractures, using functional outcomes, complication rates, and patient satisfaction tools at a six-month follow-up.

Our results show that ORIF leads to significantly better functional recovery, with OMASs consistently higher in patients with ORIF than in nonoperative management. We, therefore, believe this is consistent with published literature that also suggests that ensuring adequate anatomical reduction with stable fixation accomplished with the surgical approach is likely to optimize functional outcomes as well as minimize the risk of malunion and post-traumatic arthritis [12,13]. Despite the nonoperative group having lower OMASs, the significant proportion (74%) stating satisfactory functional recovery in our study is a strong endorsement for nonoperative management of stable Weber B fractures and aligns well with previous studies supporting this strategy [14,15].

Rates of complications in both groups were consistent with previously reported data. The operative group had a low rate of surgical site infection (4.6%) and syndesmotic malreduction (1.5%); the nonoperative group, by contrast, had increasing rates of malunion (8%) and a smaller proportion (2%) progressed to post-traumatic arthritis. These findings are consistent with previous studies that observe a slight increase in the risk of complications with nonoperatively managed fractures, particularly in cases of subtle instability missed during the initial assessment [16,17]. This highlights the need for thorough radiographic and clinical evaluation, including stress testing and advanced imaging modalities, to warrant adequacy in management [18].

In the operatively treated group, 94% of patients were satisfied with the results of treatment compared to 86% of the nonoperatively treated group (*P* = 0.0001). The slightly lower satisfaction reported by those in the nonoperative group may be attributed to prolonged immobilization periods and fears of residual deformities in malunion cases. These findings are consistent with other research that also indicates similar trends in patient satisfaction for each modality of care [19,20].

This study also raises critical considerations regarding treatment selection for isolated Weber B fractures. Although surgery is generally the treatment of choice for those with unstable or utterly displaced fractures, our work suggests that we can still find pretty good outcomes with nonoperative management in carefully selected stable fractures. Recent advancements in imaging modalities, such as weight-bearing radiographs and MRI, may enhance the ability to differentiate stable from unstable fractures, thereby allowing for more individualized treatment strategies [21].

Besides its strengths, this study has a few limitations, including a short follow-up of six months, a retrospective design, and a single-center setting, which limit the generalizability of the results. Future investigations should focus on longer follow-up periods, the design of multicenter studies for better data collection, and the incorporation of patient-reported outcome measures to determine the optimal approach for managing isolated Weber B fractures.

## Conclusions

Operative management with anatomical reduction, stable fixation, and early mobility has seen increased functional recovery with a lower risk of malunion and other complications. Despite this, the nonoperative management approach of stable fractures resulted as a viable option that provided good results in the vast majority of cases. These results emphasize the value of patient-tailored treatment planning based on detailed clinical and modality analysis to achieve the best outcomes for patients.
